# The Hippo–YAP/TAZ Signaling Pathway in Intestinal Self-Renewal and Regeneration After Injury

**DOI:** 10.3389/fcell.2022.894737

**Published:** 2022-07-19

**Authors:** Feihong Deng, Zengrong Wu, Fei Zou, Su Wang, Xuehong Wang

**Affiliations:** ^1^ Department of Gastroenterology, The Second Xiangya Hospital, Central South University, Changsha, China; ^2^ Research Center of Digestive Disease, Central South University, Changsha, China

**Keywords:** hippo–YAP/TAZ pathway, intestinal self-renewal, intestinal regeneration, intestinal stem cell, inflammatory bowel disease

## Abstract

The Hippo pathway and its downstream effectors, the transcriptional coactivators Yes-associated protein (YAP) and transcriptional coactivator with PDZ-binding motif (TAZ), control stem cell fate and cell proliferation and differentiation and are essential for tissue self-renewal and regeneration. YAP/TAZ are the core components of the Hippo pathway and they coregulate transcription when localized in the nucleus. The intestinal epithelium undergoes well-regulated self-renewal and regeneration programs to maintain the structural and functional integrity of the epithelial barrier. This prevents luminal pathogen attack, and facilitates daily nutrient absorption and immune balance. Inflammatory bowel disease (IBD) is characterized by chronic relapsing inflammation of the entire digestive tract. Impaired mucosal healing is a prominent biological feature of IBD. Intestinal self-renewal is primarily dependent on functional intestinal stem cells (ISCs), especially Lgr5^+^ crypt base columnar (CBC) cells and transient-amplifying (TA) cells in the crypt base. However, intestinal wound healing is a complicated process that is often associated with epithelial cells, and mesenchymal and immune cells in the mucosal microenvironment. Upon intestinal injury, nonproliferative cells rapidly migrate towards the wound bed to reseal the damaged epithelium, which is followed by cell proliferation and differentiation. YAP is generally localized in the nucleus of Lgr5^+^ CBC cells, where it transcriptionally regulates the expression of the ISC marker Lgr5 and plays an important role in intestinal self-renewal. YAP/TAZ are the primary mechanical sensors of the cellular microenvironment. Their functions include expanding progenitor and stem cell populations, reprogramming differentiated cells into a primitive state, and mediating the regenerative function of reserve stem cells. Thus, YAP/TAZ play extremely crucial roles in epithelial repair after damage. This review provides an overview of the Hippo–YAP/TAZ signaling pathway and the processes of intestinal self-renewal and regeneration. In particular, we summarize the roles of YAP/TAZ in the phases of intestinal self-renewal and regeneration to suggest a potential strategy for IBD treatment.

## Introduction

The Hippo signaling pathway is important in organ development, tissue growth, and tumorigenesis (Barry and Camargo, 2013; Johnson and Halder, 2014; Meng et al., 2016). Yes-associated protein (YAP) and transcriptional coactivator with PDZ-binding motif (TAZ) are the core components of the Hippo pathway and they generally act as transcriptional coactivators to initiate the transcription of target genes after binding other transcription factors in the nucleus (Johnson and Halder, 2014; Piccolo et al., 2014). The cellular localization of YAP/TAZ is particularly essential for their activation. When the Hippo pathway is stimulated, the activated LATS1/2 kinases phosphorylate YAP/TAZ, which leads to YAP/TAZ cytoplasmic retention and degradation by the ubiquitin ligase β-TrCP. However, when Hippo signaling is inactive, the inactivated LATS1/2 kinases cannot phosphorylate YAP/TAZ, and therefore these factors translocate to the nucleus to coregulate transcription (Varelas et al., 2010; Meng et al., 2016; Deng et al., 2018). An increasing number of recent studies have investigated whether Hippo–YAP/TAZ signaling plays a pivotal role in tissue homeostasis and wound healing (Fang et al., 2018; Li et al., 2020; Qu et al., 2021).

The intact intestinal epithelial barrier represents the first line of defense against pathogens, and prevents luminal antigens from entering the mucosa and commensal bacteria translocation with subsequent immune cell activation (Camargo et al., 2007). Under normal conditions, the intestinal barrier primarily consists of enterocytes that are connected by tight junctions. All intestinal epithelial cells (IECs) in the epithelial lining are derived from intestinal stem cells (ISCs) that normally reside at the base of intestinal crypts (Yu et al., 2015a). There are two distinct ISC populations in the crypt: the first are crypt base columnar (CBC) cells marked with Lgr5, which are active cycling stem cells that mediate homeostatic self-renewal (Date and Sato, 2015); and the second are +4 cells, which are quiescent stem cells that mediate injury-induced regeneration (Tian et al., 2011). Under homeostatic conditions, the epithelial lining in the small intestine is composed of crypts and villi. ISCs first migrate towards the crypt axis and differentiate into transit-amplifying (TA) cells. They then further differentiate into absorptive cells and secretory cells, such as goblet cells and enteroendocrine cells (van der Flier and Clevers, 2009). There are no villi in the colon mucosa, and all cell types of the epithelial lining may be derived from ISCs at the base of crypts (Yu et al., 2015a). IECs undergo self-renewal every 3–5 days (Barker et al., 2008), but the repair process involves the epithelium itself and mesenchymal and immune cells (Neurath, 2014; Taniguchi et al., 2015). Approximately 7 days is generally needed for complete regeneration (Sprangers et al., 2021). Ulcerative colitis (UC), which is a subcategory of inflammatory bowel disease (IBD), is characterized by chronic relapsing inflammation of the colon (Lasry et al., 2016; Deng et al., 2018). Two prominent pathophysiological features of UC are dysregulation of the immune system and impaired epithelial regeneration (Krishnan et al., 2011). Mucosal healing is a key treatment goal for UC (Pineton de Chambrun et al., 2010; Neurath and Travis, 2012).

YAP/TAZ are particularly essential for intestinal homeostasis and epithelial regeneration (Cai et al., 2010; Barry and Camargo, 2013; Imada et al., 2016; Deng et al., 2018; Dey et al., 2020). Systematic overexpression of YAP in mice has accelerated colonic self-renewal, and led to an increased number of proliferating crypt cells and the activation of cell migration along the crypt axis, as determined by BrdU labeling (Deng et al., 2018). YAP mediates the function of Src family kinases (SFKs) in the proliferation of IECs within intestinal crypts and contributes to the proper regulation of intestinal homeostasis (Imada et al., 2016). YAP knockout or YAP/TAZ double-knockout mice treated with dextran sulfate sodium (DSS) or irradiation have shown diminished intestinal epithelium regeneration and extensive crypt loss (Gregorieff et al., 2015). YAP-deficient mice exhibited a dramatic decrease in crypt proliferation, which was accompanied by the clear downregulation of the ISC marker Olfm4 (Wang et al., 2017). The present review provides a broad overview of the Hippo–YAP/TAZ signaling pathway, and the role of YAP/TAZ in intestinal homeostasis and epithelial regeneration.

### Overview of the Hippo–YAP/TAZ Signaling Pathway

**FIGURE 1:**
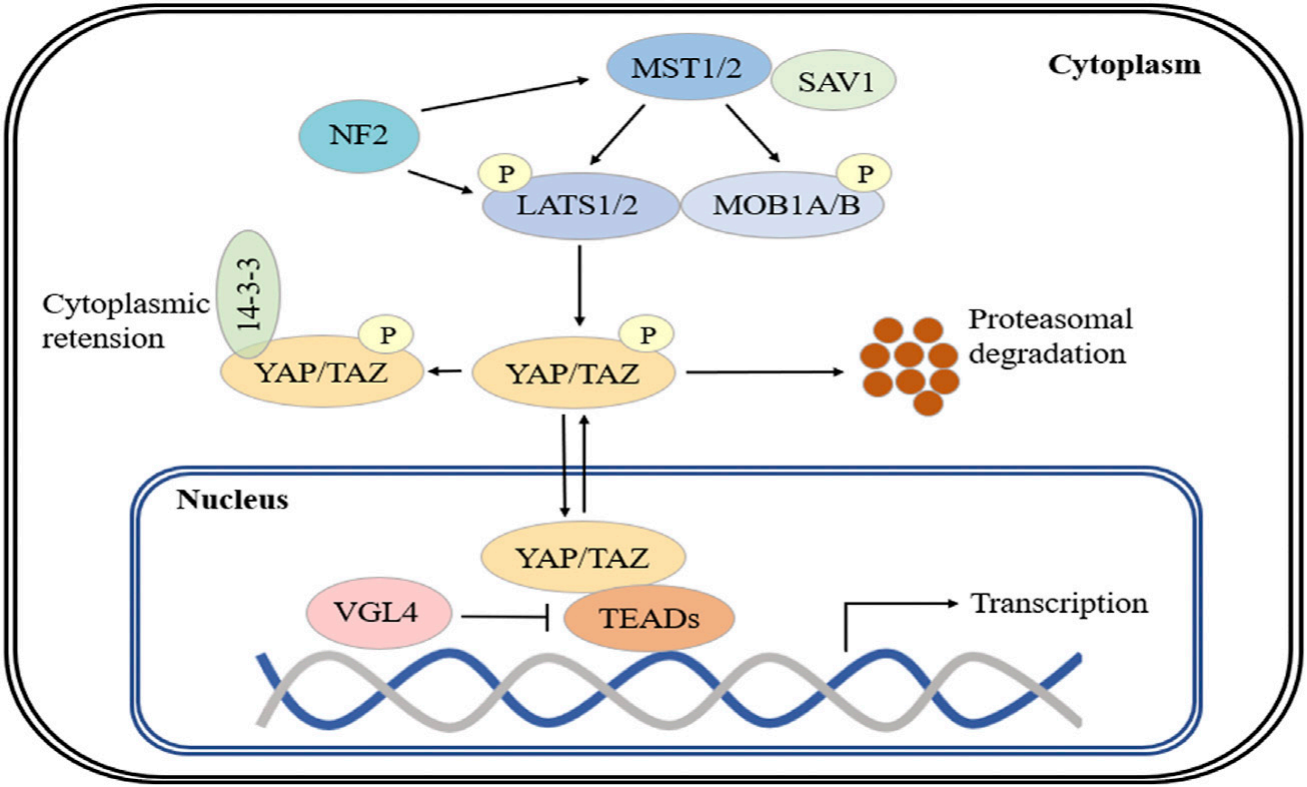
The Hippo signaling pathway. Major mammalian Hippo signaling pathway components. When the Hippo pathway is active, MST1/2 activates and phosphorylates MOB1A/B and LATS1/2, and activated LATS1/2 phosphorylates YAP and TAZ. Phosphorylated YAP/TAZ is sequestered in the cytoplasm by binding to 14-3-3 protein and is degraded by β-TrCP. In the absence of Hippo signaling, inactive LATS1/2 cannot phosphorylate YAP/TAZ, and YAP/TAZ translocate to the nucleus and interact with TEAD transcription factors to promote target gene expression. VGL4 competes with YAP for TEAD binding. NF2 directly interacts with LATS1/2 and induces its phosphorylation.

The Hippo pathway is an important regulator of organ development and tissue growth in *Drosophila melanogaster*, and is highly conserved in mammals (Johnson and Halder, 2014; Yu et al., 2015b; Wang et al., 2017). The core components of the Hippo pathway include the MST1/2 and LATS1/2 kinases and their respective scaffolding proteins, SAV1 (Salvador) and MOB1A/B (Varelas et al., 2010; Meng et al., 2016). Generally, MST1/2 phosphorylates LATS1/2, which results in the activation of LATS1/2. Activated LATS1/2 phosphorylates the transcriptional coactivators YAP and TAZ, which inactivates these factors and causes their retention in the cytoplasm (Johnson and Halder, 2014; Yu et al., 2015b). Another key regulator of LATS1/2 phosphorylation is NF2, which directly interacts with LATS1/2 and induces phosphorylation in an MST-independent manner (Wang et al., 2017). YAP phosphorylation at serine 127 (S127) and TAZ phosphorylation at serine 89 (S89) induce their binding to 14-3-3 proteins and cytoplasmic sequestration. LATS1/2 induces YAP phosphorylation at S381 (TAZ phosphorylation at S311) by CK1ε and -δ and coordinated recruitment of the ubiquitin ligase β-TrCP, which evokes YAP/TAZ ubiquitination and degradation (Koo and Guan, 2018). When Hippo is inactive, LATS1/2 cannot phosphorylate YAP/TAZ, which allows these coactivators to translocate to the nucleus and interact with other transcription factors to induce transcription (Yu et al., 2015b; Totaro et al., 2018). YAP/TAZ lack a DNA-binding domain and must bind with other transcription factors (e.g., TEADs, SMADs, TBX, RUNX and p73) to form transcriptional complexes that are capable of regulating gene expression (Johnson and Halder, 2014). Notably, VGL4 competes with YAP for TEAD binding, which results in the suppression of target gene transcription (Yu et al., 2015b; Wang et al., 2017) (Figure 1).

The activity of the Hippo pathway is regulated by various signals and factors, including physical cues such as cell contact and mechanical signals, regulators upstream of MST kinases, G protein-coupled receptors (GPCRs) and adherens junctions (Boggiano and Fehon, 2012). YAP is primarily expressed in Lgr5^+^ CBC cells in intestinal crypts (Yu et al., 2015a). Meanwhile, elevated YAP activity is associated with stem and progenitor cell expansion coupled with suppressed cell differentiation (Johnson and Halder, 2014). This suggests that there is a role for YAP in regulating ISC function and intestinal homeostasis. Notably, YAP shows predominant nuclear localization and enhanced expression in the epithelium following chemical- and irradiation-induced injury (Gregorieff et al., 2015; Deng et al., 2018). This reveals its crucial effects on epithelial cell proliferation and mucosal healing.

### The Process of Intestinal Self-Renewal

The intestinal epithelium is one of the most self-renewing mammalian tissues (Barker et al., 2008). In the small intestine, Lgr5^+^ ISCs divide every 24 h to generate highly proliferative TA cells. These progeny reside in the crypt for approximately 2 days, during which they undergo four to five cell cycles before terminally differentiating into various cell lineages of intestinal villi, including absorptive enterocytes, goblet cells, enteroendocrine cells, and Paneth cells (Li and Clevers, 2010; Clevers, 2013). After terminal differentiation, the mature cells reach the tip of the villus, undergo programmed cell death, and are then shed into the intestinal cavity. A new generation of TA cells then proliferate, migrate, and replace the dead cells in a new self-renewal cycle (van der Flier and Clevers, 2009; Yu et al., 2015a). The colonic epithelium lacks villi but is organized similarly, with differentiated cells, including colonocytes and goblet cells, populating the flattened upper regions of the crypts (Barker et al., 2007; Barker et al., 2012). Although Paneth cells are absent in the colon epithelium, deep secretory cells (DSCs) perform equivalent functions (Sasaki et al., 2016). DSCs are intercalated between CBC cells at the bottom of the crypt and are marked by REG4 (Sasaki et al., 2016; Beumer and Clevers, 2021). Ablation of DSCs has been shown to induce the loss of Lgr5^+^ stem cells from colonic crypts, and has hampered gut homeostasis and colon organoid growth (Sasaki et al., 2016). The small intestinal and colonic epithelium display a similarly high self-renewal rate: during self-renewal, highly proliferative TA cells originate from ISCs, migrate along the crypt, and undergo differentiation when they reach the luminal surface (Karam, 1999; de Santa Barbara et al., 2003; Onfroy-Roy et al., 2020). Normal intestinal self-renewal is important for maintaining the integrity of the epithelial barrier, which contributes to daily nutrient absorption and immune balance.

**FIGURE 2:**
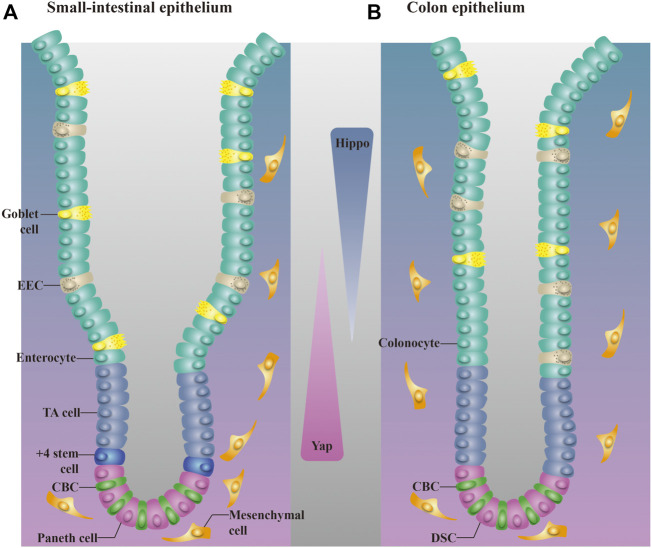
Distribution of different cells in the small intestinal epithelium and colon epithelium. **(A)**. The small intestinal epithelium is organized into crypts and villi. CBC cells are present among Paneth cells, and +4 cells are restricted to the +4 position above the Paneth cell. TA cells are located above +4 cells. **(B)**. The colon epithelium lacks villi. Most cell types in the colon epithelium are also found in the small intestine. DSCs are equivalent to Paneth cells in the colon and are intercalated between CBC cells. EEC, enteroendocrine cell. Activation of the Hippo pathway follows an ascending gradient along the crypt–villus axis, whereas YAP activity shows a descending trend.

Two distinct ISC populations coexist in the intestinal crypt: Lgr5^+^ CBC cells, which are present among Paneth cells, and Bmil^+^ +4 cells, which are mostly restricted to the “+4” cell position above the Paneth cell in the proximal small intestine (Li and Clevers, 2010; Yan et al., 2012). Lgr5^+^ CBC cells are important in homeostatic self-renewal (Date and Sato, 2015) and are indispensable for the repair of damaged intestine. Chemical- or irradiation-induced epithelial injury in mice always manifests as the absence of Lgr5^+^ ISCs in crypts (Tao et al., 2015; Girish et al., 2021), but the intestinal epithelium recovers (Metcalfe et al., 2014). Bmil^+^ +4 cells are responsible for injury-induced regeneration and they represent a reserve stem cell (RSC) pool upon injury of the intestine epithelium (Tian et al., 2011). Normally quiescent Bmi1^+^ ISCs undergo dramatic proliferation to clonally repopulate multiple contiguous crypts and villi after irradiation (Yan et al., 2012). These two cell populations are also closely connected: Bmil^+^ +4 cells produce Lgr5^+^ CBC cells in intestinal crypts under injury and nonpathological conditions (Sangiorgi and Capecchi, 2008; Tian et al., 2011; Date and Sato, 2015). Paneth cells drive the formation of new stem cells and are critical for the maintenance of CBC cells (Sato et al., 2011). Genetic deletion of Paneth cells has been shown to result in the consistent absence of Lgr5^+^ ISCs in crypts (Sato et al., 2011). Moreover, mice lacking Paneth cells maintained an almost normal epithelial structure for more than 6 months (Bastide et al., 2007), which suggests that the intestine functions normally in the absence of Lgr5^+^ ISCs, and that +4 cells may reconstitute the Lgr5^+^ ISC compartment and help preserve intestinal homeostasis (Figure 2).

### Intestinal Self-Renewal Regulated by YAP/TAZ

Lgr5^+^ CBC cells residing in crypts divide into highly proliferative TA cells that terminally differentiate into specific cell lineages of the intestine, including absorptive enterocytes and secretory cells (Yu et al., 2015a). Along the crypt–villus axis, YAP is predominantly located in the cytoplasm of TA cells (Zhou et al., 2011) but can also be found in the nucleus of Lgr5^+^ CBC cells in the crypt (Barry et al., 2013). This creates a descending gradient of Hippo activity (ascending YAP activity) from the villus to the crypt base. Enhanced YAP or TAZ activity is particularly associated with stem and progenitor cell expansion, which is accompanied by the inhibition of differentiation (Johnson and Halder, 2014). These processes have been investigated in mammalian stem and progenitor cells of the intestine, liver, pancreas, heart, and skin (Camargo et al., 2007; Schlegelmilch et al., 2011; Heallen et al., 2011; von Gise et al., 2012; Yan et al., 2022). In murine embryonic stem cells, YAP directly binds to the promoters of multiple genes that modulate pluripotency (Lian et al., 2010). This indicates that YAP plays a critical role in maintaining stem cell pluripotency. This hypothesis is also supported by the findings that YAP and TAZ double-knockout embryos died before the morula stage (16–32 cells) (Nishioka et al., 2009), and that YAP overexpression or LATS2 deletion enhanced the reprogramming of differentiated cells into pluripotent stem cells (Qin et al., 2012). The continuous division of progenitor TA cells in the crypt drives the migration of existing mature IECs along the crypt and is regarded as a major determinant of the self-renewal rate of mature IECs (Clevers, 2013). Using a mouse model of stem cell-based incisor renewal, Hsien Hu et al. found that YAP and TAZ were required to maintain proliferation and prevent premature differentiation of TA cells. FAK activates YAP signaling and induces YAP nuclear localization with subsequent activation of mTOR signaling, thereby promoting TA cell proliferation. These data indicate that YAP/TAZ signaling coordinates stem cell expansion and differentiation during tissue renewal (Hu et al., 2017).

Lgr5^+^ ISCs require a crypt niche that is derived from Paneth cells and stromal cells underneath the epithelial lining (Kabiri et al., 2014). The function of the stem cell niche is regulated by multiple signaling pathways, such as the Wnt, BMP and Notch pathways (Yu et al., 2015a). Paneth cells are the source of Wnt, which drives the formation of new stem cells. Wnt signals also drive the formation of new Paneth cells (Clevers, 2013). This process is dependent on the transcription factor Sox9, which is regulated by the Wnt/TCF complex (Bastide et al., 2007). In addition, this Wnt-driven positive feedback loop may result in ever-expanding intestinal crypts (Clevers, 2013). Wnt signaling is crucial in intestinal homeostasis (Kretzschmar and Clevers, 2017; Han et al., 2020). In the absence of Wnt, cytoplasmic β-catenin is recruited to the Axin/APC/GSK3/CK1 destruction complex, it is consequently phosphorylated by GSK3 and CK1 and degraded by β-TrCP. When Wnt is present, β-catenin escapes degradation by the destruction complex, translocates into the nucleus, and interacts with TCF/LEF to initiate transcription (Imajo et al., 2012). Lgr5 and Axin2 are putative ISC markers and typical target genes of Wnt signaling (Boonekamp et al., 2021). YAP localizes in the nucleus of Lgr5^+^ CBC cells within the crypt base under homeostatic conditions. Meanwhile, systematic overexpression of YAP promotes epithelial cell proliferation and migration along crypts to accelerate self-renewal.

Numerous recent studies have focused on the regulatory mechanism between YAP/TAZ and Wnt signaling because of their overlapping roles in stem cell regulation and intestinal homeostasis. In Paneth/goblet-like cells of the colon, Pla2g2a inhibits Wnt signaling by increasing YAP phosphorylation to negatively regulate the ability of ISCs to form organoids (Schewe et al., 2016). Phosphorylation of YAP at Ser127 induces cytoplasmic translocation and is required for the suppression of β-catenin/TCF-mediated transcription (Imajo et al., 2012). TAZ degradation depends on the GSK3-mediated phosphorylation of β-catenin, which bridges TAZ to β-TrCP, whereas β-catenin release from the destruction complex hampers TAZ degradation and leads to the concomitant nuclear accumulation of β-catenin and TAZ (Azzolin et al., 2012). YAP and TAZ constitute the β-catenin destruction complex when Wnt signaling is inactive, and YAP/TAZ directly interact with Axin to mediate the recruitment of β-TrCP and the inactivation of YAP/TAZ and β-catenin. Upon Wnt activation, YAP/TAZ are released from the destruction complex. This leads to YAP/TAZ nuclear localization and the activation of Wnt/β-catenin-mediated transcription (Azzolin et al., 2014). Notably, β-catenin-driven transcription is dependent on the presence of YAP (Rosenbluh et al., 2012), and Lgr5 is a transcriptional target of the YAP/β-catenin/TCF4 complex (Deng et al., 2018). These data indicate that YAP mediates β-catenin-driven transcription to regulate ISC marker expression during intestinal homeostasis. However, further in-depth research into the roles of YAP/TAZ and Wnt signaling in intestinal self-renewal may be needed.

### The Process of Mucosal Healing in the Gut

The mucosal repair process represents a series of complicated physiological events that involve multiple factors, including epithelial cells, growth factors, stem cells, and inflammatory cells (Kurashima and Kiyono, 2017). There are two crucial steps for mucosal healing: reducing the mucosal inflammation burden and targeting the epithelium to promote tissue regeneration. The epithelium shows marked neutrophil accumulation in the early phase of the inflammatory process (day 1) in the damaged gut (Rieder et al., 2007; Weidenbusch and Anders, 2012). In this phase, the mucosal environment is dominated by pathogen-associated molecular patterns (PAMPs) from microorganisms and from damage-associated molecular patterns (DAMPs) from dying cells (Weidenbusch and Anders, 2012). PAMPs and DAMPs activate innate pattern recognition receptors on immune cells, which activate infiltrating leukocytes and induce the inflammatory phenotype (Rock et al., 2010). In this PAMP- and/or DAMP-rich environment, there is an increased presence of macrophages at the injury site. These infiltrating macrophages adopt the classically activated phenotype (M1) and secrete proinflammatory cytokines, including TNF-α, IL-1, IL-6, and IL-23 (Mantovani et al., 2004; Galli et al., 2011). However, this process is sustained for only 2–3 days (Swaminathan and Griffin, 2008) and is followed by a decrease in DAMPs and PAMPs, and an increase in the number of apoptotic neutrophils. This favors macrophage polarization towards an anti-inflammatory phenotype (M2) and the release of anti-inflammatory cytokines, such as TGF-β and IL-10, to support a reduction in mucosal inflammation (Lucas et al., 2003; Deng et al., 2019). During the acute phase, neutrophils diminish after the first 1–3 days but continue to reside at wound sites under conditions of chronic inflammation because of their continuous recruitment in response to bacterial invasion (Diegelmann, 2003; Leoni et al., 2015). Therefore, inflammation is a crucial component of the mucosal healing process and uncontrolled inflammation limits subsequent tissue regeneration.

Fibroblasts and myofibroblasts accumulate and proliferate at the wound bed in the epithelium regeneration process. These cells express smooth muscle actin (SMA) and produce abundant collagen to initiate mucosal repair (Leoni et al., 2015; Kurashima and Kiyono, 2017). This collagen-rich matrix replaces the provisional damaged matrix, and fibroblasts and myofibroblasts migrate into the wound sites to remodel the extracellular matrix (ECM) (Leoni et al., 2015). The synthesis of ECM by fibroblasts at injury sites is modulated by TGF-β and other proteins, such as IL-1, IL-4, and vascular endothelial growth factor (VEGF) (Leoni et al., 2015). Consistent with reconstruction of the ECM, epithelial restitution simultaneously occurs (Kurashima and Kiyono, 2017). During epithelial restitution, nonproliferative cells from neighboring crypts, which are called wound-associated epithelial (WAE) cells, rapidly migrate over the wound bed to reseal the damaged epithelium. This process generally occurs within minutes to hours after injury (Miyoshi et al., 2012; Sprangers et al., 2021) and is key for preserving or reestablishing the mucosal barrier as a host defense system. No cell proliferation is involved in this phase, which is primarily controlled by cytokines, such as TGF-β, KGF, and TFFs, in the mucosal microenvironment (Sturm and Dignass, 2008; Krishnan et al., 2011). TGF-β produced by mesenchymal cells induces the conversion of fibroblasts to myofibroblasts and stimulates myofibroblasts located beneath IECs to produce multidomain ECM proteins, which initiate the movement of IECs to cover damaged mucosal surfaces (Kurashima and Kiyono, 2017). TFF3 prevents epithelial cell apoptosis and promotes epithelial cell migration to cover epithelial wounds (Mashimo et al., 1996). Therefore, epithelial migration requires the dynamic and coordinated reconstruction of cell–cell and cell–matrix adhesions. Epithelial cells at the leading edge extrude filamentous actin-rich protrusions that dynamically adhere to the matrix and mediate the forwards migration of epithelial cells (Yang et al., 2012). Therefore, the rapid activation of collagen-expressing mesenchymal cells and the induction of well-regulated epithelial restitution are extremely important for the initial reestablishment of the mucosal barrier.

After epithelial restitution, rapid cell proliferation is triggered and RSCs (primarily +4 cells) dramatically proliferate into large regenerating crypts (Sprangers et al., 2021). Because Lgr5^+^ CBC cells are almost diminished upon chemical- or irradiation-induced injury (Girish et al., 2021), +4 cells, as progenitors of the absorptive and secretory lineages, are reactivated by the mucosal damage and contribute to the epithelial regenerative response by restoring the Lgr5^+^ stem cell pool (Tian et al., 2011). Musashi-1 and DCAMKL1 are recognized markers of +4 cells. Significantly increased expression of these markers was detected during the healing or regenerative phase in murine colitis models despite an initial decrease in expression after injury. However, no significant change in Lgr5 expression was observed in these models. This indicates the critical role of +4 cells, rather than Lgr5^+^ ISCs, in injury-induced regeneration (Fukui et al., 2006; Dekaney et al., 2009). Other research has shown that Lgr5^+^ CBC cells are indispensable for intestinal repair and provide the emergency replenishment of TAs and ISCs upon injury, whereas quiescent +4 cells divide infrequently and preserve long-term quiescence (Wang and Hou, 2010).

**FIGURE 3:**
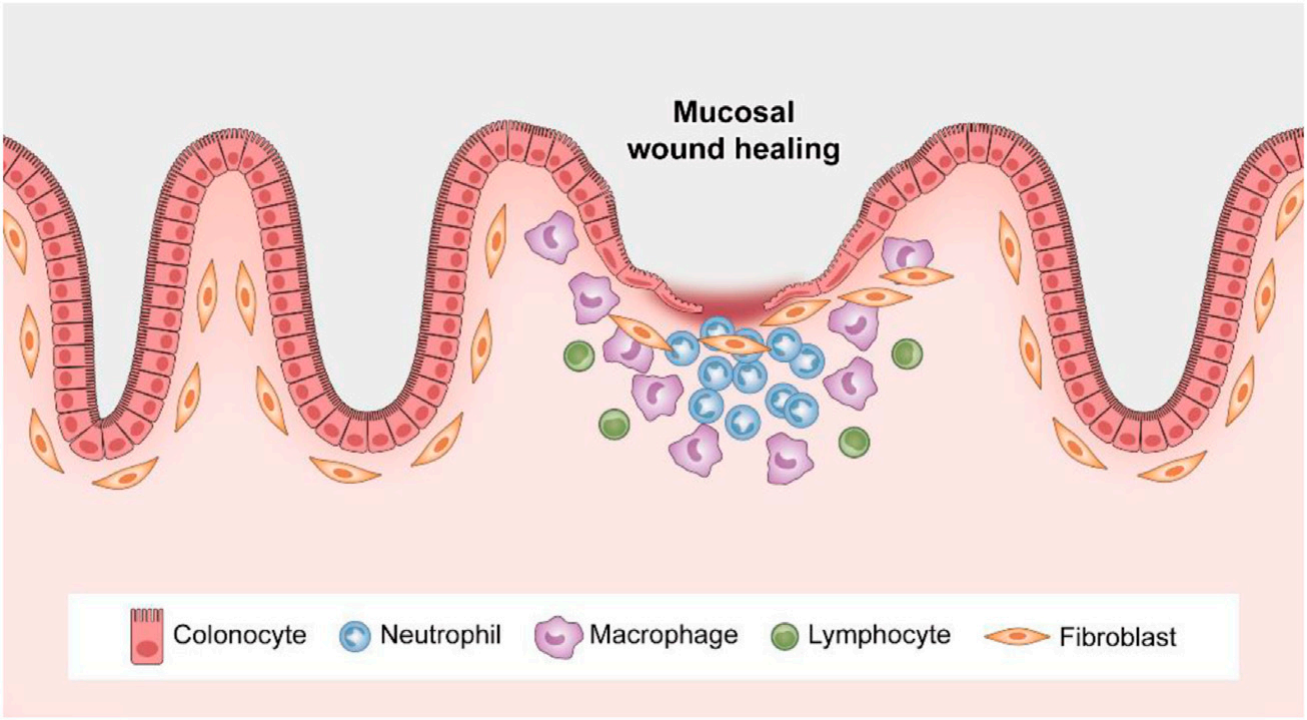
Spatiotemporal control of wound repair. After mucosal injury, neutrophils rapidly accumulate at the wound site, followed by an increased presence of macrophages. Epithelial restitution is achieved by epithelial cell migration and subsequent proliferation.

The cell proliferation phase generally lasts until 4 days after injury (Kim et al., 2017). Recent novel findings show that regeneration of the damaged epithelium requires the transient reprogramming of epithelial cells into a fetal-like state (Nusse et al., 2018; Yui et al., 2018; Ayyaz et al., 2019). The epithelium undergoes a dedifferentiation program in which adult stem cell and differentiated cell markers (such as Lgr5) are suppressed and a fetal gene signature (which is characterized by factors such as Sca1) is correspondingly increased (Yui et al., 2018; Sprangers et al., 2021). Notably, the cell proliferation process involves the epithelial cells themselves, regulatory factors produced by the underlying mesenchyme, and recruited inflammatory cells in the local gut microenvironment, including macrophages, endothelial cells and dendritic cells (Pull et al., 2005; Seno et al., 2009). This suggests the importance of internal epithelial cell–epithelial cell and epithelial cell–immune cell crosstalk in mucosal regeneration. Antimicrobial peptides released by Paneth cells and mucins produced by goblet cells also participate in this process, which prevents the translocation of commensal bacteria and subsequent immune activation (Kurashima and Kiyono, 2017). A normalization phase follows until day 7 postinjury, during which crypt structure and number recover (Sprangers et al., 2021). Taken together, the data indicate that while reducing mucosal inflammation, the well-controlled processes of ECM remodeling, epithelial restitution, cell proliferation and differentiation ultimately lead to closure of mucosal erosions and ulcerations (Figure 3).

### Intestinal Regeneration Regulated by YAP/TAZ

Tissue regeneration is an intricate process that is initially triggered by marked changes in the environment at the cellular and biophysical levels (Stappenbeck and Miyoshi, 2009). YAP and TAZ were recently recognized as primary sensors of the cellular microenvironment (Piccolo et al., 2014) that integrate cell polarity and physical cues with growth factors and inflammation. After surface injury, the loss of adherens junctions and tight junctions between epithelial cells results in the inactivation of LATS and the activation of YAP and TAZ (Levy et al., 2008). Growth factors and cytokines regulate the production of collagen-rich matrix by fibroblasts at the injury sites to rapidly replace the damaged matrix and induce ECM reconstruction (Leoni et al., 2015). The physical attachment of epithelial cells to the ECM is essential for cells to survive and reseal the wound bed (Meng et al., 2016). YAP is active in this repair process, which shows nuclear localization in response to activation by Rho GTPases or the FAK–Src–PI3K pathway (Zhao et al., 2012; Kim and Gumbiner, 2015). Disruption of F-actin inhibits the effect of attachment on YAP phosphorylation and nuclear localization (Meng et al., 2016). Rho GTPases regulate YAP/TAZ activity. Rac1 is a member of the Rho GTPase family and controls cellular protrusions at the leading edge (Yamaguchi et al., 2015). Rac1 expression results in epithelial movement and proliferation by targeting β1 integrin in cellular protrusions and modulating actin dynamics (Yamaguchi et al., 2015). Animals treated with FAK and Src inhibitors showed an inability to repair large ulcerations (Yui et al., 2018). This demonstrates that cell mechanics regulated by the Rho GTPase/YAP and FAK/YAP signaling pathways facilitate epithelial cell migration, and are crucial for resealing the injured epithelial surface and repairing the gut mucosa. These results indicate that YAP and TAZ, as the primary mechanical sensors of the cellular microenvironment, functionally participate in ECM reconstruction and epithelial restitution to facilitate mucosal healing by regulating different signaling pathways.

Tissue repair and regeneration always involve stem cell activation and progenitor cell expansion (Cosín-Roger et al., 2013). Enhanced YAP and/or TAZ activity is associated with stem and progenitor cell expansion, coupled with the inhibition of differentiation (Johnson and Halder, 2014). The Notch and Wnt signaling pathways are crucial for the maintenance of ISCs in the undifferentiated, proliferative state (van der Flier and Clevers, 2009; Neal et al., 2011). The Notch pathway primarily regulates absorptive versus secretory fate decisions in the intestinal epithelium (Camargo et al., 2007). Inhibition of the Notch pathway in the intestinal epithelium results in the rapid and complete conversion of all epithelial cells into goblet cells (Milano et al., 2004). The intestinal epithelium in Hes1 knockout models exhibited increases in Paneth, goblet, and enteroendocrine cells and a decrease in absorptive enterocytes (Suzuki et al., 2005). Notably, Notch signaling generally acts downstream of YAP, and YAP-mediated intestinal progenitor/stem cell expansion is at least partially associated with activation of the Notch signaling pathway (Camargo et al., 2007). Inhibition of Notch signaling suppresses YAP-induced intestinal proliferation (Camargo et al., 2007).

In addition to stem/progenitor cell expansion in crypts, reprogramming of differentiated cells into a primitive fetal-like state in the epithelium is required for epithelial regeneration (Nusse et al., 2018; Yui et al., 2018; Ayyaz et al., 2019). Upon tissue damage, cells other than adult ISCs are the major contributors to wound repair (Blanpain and Fuchs, 2014). In a DSS-induced colonic regeneration mouse model, the epithelium showed transient reprogramming characterized by the *de novo* expression of fetal markers, and the suppression of adult stem cell and differentiated cell markers, such as Lgr5, Olfm4, and Lrig1 (Barker et al., 2012; Yui et al., 2018). Activation of YAP/TAZ reprograms differentiated cells to a stem and progenitor cell state (Yui et al., 2018). Intestinal organoids in a regenerative state show a fetal signature and highly uniform nuclear YAP expression (Sprangers et al., 2021). Sca1 is a cellular marker of the fetal state and a characteristic marker of epithelium repair. A number of adult stem cell markers have been reported to be specifically inhibited in a Sca1^high^ state (Yui et al., 2018). YAP and TAZ are required for the repair process after DSS-induced injury and are closely associated with driving the formation of the Sca1-expressing repaired epithelium (Yui et al., 2018). YAP overexpression has been noted to increase markers of the fetal epithelium, and decrease markers related to adult stem cells and differentiated lineages (Yui et al., 2018). Notably, the cell fate changes that are associated with injury-induced reprogramming were reversible *in vivo* and *in vitro*, which allowed the tissue to regain normal cellular structure once epithelial regeneration was complete (Deng et al., 2018). Therefore, YAP/TAZ promote the reprogramming of differentiated cells into a more primitive state, and the expansion of progenitor/stem cells is important in epithelial regeneration. These findings may suggest prospective strategies for the development of regenerative medicine.

**FIGURE 4:**
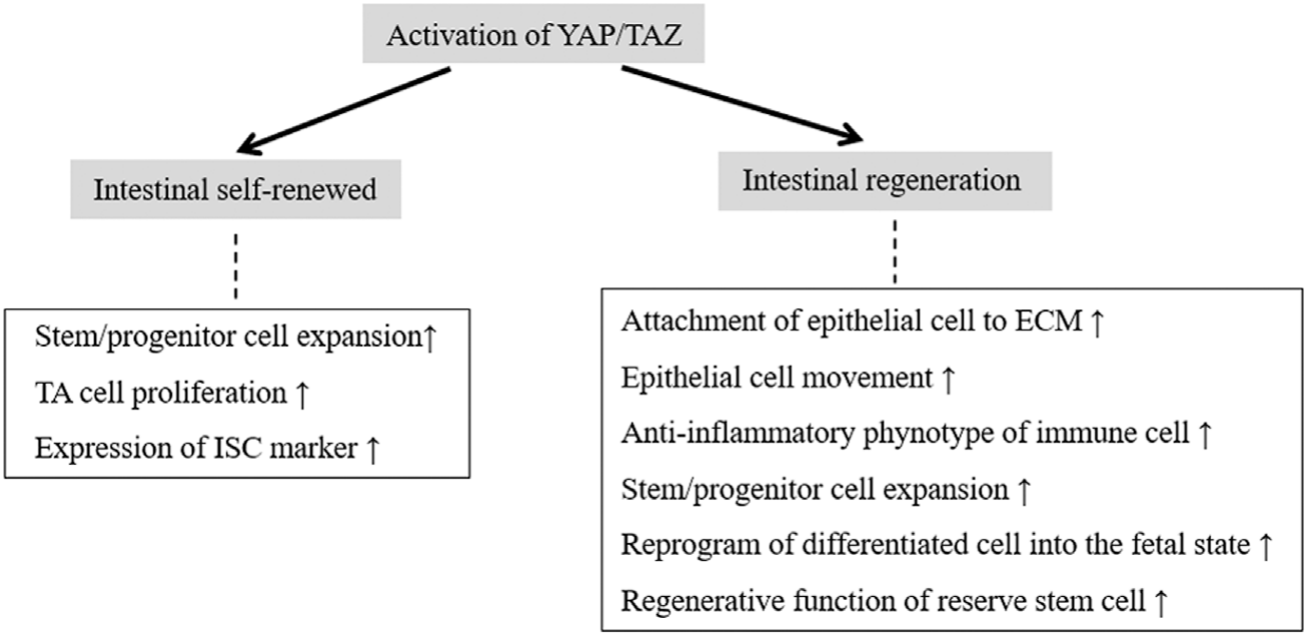
The roles of YAP/TAZ activation in intestinal self-renewal and regeneration.

Lgr5^+^ stem cells are the multipotent cell source in regeneration. This cell population is almost absent following irradiation, despite the lack of obvious abnormalities in intestinal architecture (Tao et al., 2015). This suggests that an additional population of RSCs (e.g., +4 cells) can replenish the loss of Lgr5^+^ ISCs or that postmitotic differentiated cells have the potential to revert to a stem cell state. Normally quiescent +4 cells, or RSCs, that reside above Paneth cells in the crypt base play a profound role in injury-induced regeneration (Li and Clevers, 2010; Tian et al., 2011). Progenitors of the secretory and absorptive lineages and quiescent +4 cells act as RSC pools that are reactivated after the injury-induced depletion of Lgr5^+^ ISCs and give rise to fully functional Lgr5^+^ stem cells that help to re-establish the epithelial barrier (Bankaitis et al., 2018; Hageman et al., 2020). Overexpression of YAP activates β-catenin/TCF4-driven transcription of Lgr5 in epithelial cells and markedly accelerates colonic regeneration after DSS-induced colitis (Deng et al., 2018). YAP mediates the regenerative function of revival stem cells (revSCs), which are extremely rare under homeostatic conditions but are mobilized upon injury (Ayyaz et al., 2019). After intestinal damage by irradiation or DSS, which ablates Lgr5^+^ CBC cells in the crypt base, revSCs undergo YAP-dependent transient expansion to reconstitute the Lgr5^+^ CBC compartment and promote effective intestinal regeneration (Ayyaz et al., 2019). This indicates that +4 cells or RSCs are partially dependent on YAP to perform their function in injury-induced intestinal repair (Figure 4).

Dynamic changes in YAP expression are observed during the epithelial repair process, in which YAP protein levels gradually increase in the early stages of regeneration but return to normal once the intestinal structure is fully restored (Yu et al., 2015a). Notably, switching of YAP from the active to the inactive state may represent a key event that initiates the normalization phase of injury repair *in vivo*. As a potential link between YAP and Wnt signaling regulation in epithelial regeneration, YAP transiently reprograms Lgr5^+^ ISCs by inhibiting Wnt signaling to induce a regenerative program (Gregorieff et al., 2015). This suggests that YAP negatively regulates the activation of Wnt signaling. In contrast to the progressive increase in YAP expression in the early process of regeneration, decreased Wnt signaling activity has been noted after early epithelial damage and was found to rapidly normalize along with recovered Lgr5 and Olfm4 expression 3 ***–*** 5 days after injury (Gregorieff et al., 2015; Ayyaz et al., 2019). The immune cells in the mucosal microenvironment are also important in the repair process phase of epithelial regeneration. Macrophages are necessary for proper colonic epithelial repair, particularly due to their function as mobile cellular transceivers that coordinate luminal microbes and the injured epithelium, and transmit regenerative signals to neighboring colonic epithelial progenitors (ColEPs) (Pull et al., 2005). Our previous study found that M2 macrophage-derived exosomal miR-590-3p could be transferred into epithelial cells and attenuate DSS-induced mucosal damage by promoting epithelial repair via the LATS1/YAP/β-catenin signaling axis (Deng et al., 2021). Abundant research has demonstrated that ILC3-driven epithelial proliferation and intestinal regeneration are dependent on STAT3/IL-22 (Zeng et al., 2019; Talbot et al., 2020). Romera-Hernández et al. (2020) reported that ILC3-driven intestinal repair and the prevention of excessive pathology are independent of STAT3 but involve the activation of gp130/SFK/YAP signaling in intestinal crypt cells. These results demonstrate that YAP/TAZ activation is also affected by cytokines or exosomes produced by immune cells beneath the crypt base. The dynamic changes in YAP and Wnt expression during different phases of epithelial repair may be attributed to the gradual restoration of ECM architecture and the epithelial barrier, which involves the epithelium itself and the modified phenotypes of immune cells beneath the epithelial lining.

### Hippo Pathway and Inflammatory Bowel Disease

Impaired epithelial regeneration is an important biological feature of IBD (Krishnan et al., 2011) and the promotion of mucosal healing is a recent goal for clinical IBD therapy (Pineton de Chambrun et al., 2010). Mucosal healing is an intricate process that is triggered by a series of events involving the epithelium and immune and stromal cells (Taniguchi et al., 2015). Epithelial YAP is crucial for epithelial proliferation and repair (Johnson and Halder, 2014; Xie et al., 2021). As we previously reported, the overexpression of nuclear YAP in mice increases IEC proliferation and mucosal repair after DSS-induced colitis (Deng et al., 2018). Stromal cell-specific ISLR increases YAP expression in colonic epithelial cells, which accounts for the epithelial cell growth and intestinal regeneration after DSS-induced colitis (Xu et al., 2020). In nonepithelial cells, YAP is generally associated with activated mucosal inflammation. VEGF-A and TNF-α inhibit LATS1/2 activity in endothelial cells, which leads to YAP/TAZ activation, promotes the inflammatory vascular response, and increases the severity of IBD (Choi et al., 2018; Park and Kwon, 2018; Xie et al., 2021). In macrophages, YAP aggravates IBD by promoting M1 macrophage activation and suppressing M2 macrophage polarization. Deletion of YAP in murine macrophages increased the abundance of *Lactobacillus*, *Bacteroides*, and *Bifidobacterium* and decreased the abundance of *Prevotella*, β-Proteobacteria, and γ-Proteobacteria (Zhou et al., 2019). YAP/TAZ expression in fibroblasts was increased in stenotic intestines and has been associated with poor clinical outcomes in Crohn’s disease (CD) (Ou et al., 2021). Therefore, YAP functions differently in distinct cell types and overexpression of epithelial YAP may be useful for epithelial regeneration. However, YAP in nonepithelial cells is always involved in active intestinal inflammation and/or IBD aggravation. Finally, tissue- or cell-specific control of YAP/TAZ may be important for the development of new IBD therapeutics.

## Conclusion

In conclusion, the Hippo–YAP/TAZ signaling pathway appears to control stem cell fate and cell proliferation and differentiation and is important in intestinal self-renewal and regeneration. YAP/TAZ, core components of the Hippo pathway, generally act as transcriptional coactivators and initiate transcription by interacting with transcription factors. Intestinal self-renewal often occurs in less than 7 days, and is primarily dependent on actively proliferative Lgr5^+^ ISCs and TA cells in the crypt base, which is crucial for cell survival and tissue growth. YAP is predominantly located in the nucleus of Lgr5^+^ CBC cells within the gut and may help to regulate Lgr5 transcription to facilitate epithelial cell self-renewal. Notably, intestinal wound healing is a complicated process that often involves two key steps: the first step is a reduction in the mucosal inflammation burden, and the second step is the restoration of epithelial structure and function. The latter process entails well-controlled ECM remodeling, epithelial restitution, cell proliferation, and differentiation. YAP/TAZ, which are recognized as the primary sensors of the cellular microenvironment, are functionally activated during the physical attachment of epithelial cells to the ECM and epithelial cell movement. Because intestinal regeneration requires the coordination of cell proliferation and differentiation, the activation of YAP/TAZ promotes the reprogramming of differentiated cells into a fetal-like state and the expansion of progenitor/stem cells, which are important steps in epithelial regeneration after damage. YAP also partially mediates the function of RSCs in injury-induced regeneration. In IBD, YAP/TAZ expression increases during regeneration. This suggests the potential of YAP-targeted small molecules for epithelial repair in IBD. Taken together, the data indicate that YAP/TAZ regulate intestinal homeostasis and regeneration via multiple distinct mechanisms, and are essential for maintaining the structural and functional integrity of the epithelial barrier. These findings support the future development of regenerative medicine as a novel therapeutic strategy for IBD.
